# Effects of Knee Flexion Angle on Gluteus Medius Activation and Activation Ratios During the Modified Clamshell Exercise

**DOI:** 10.3390/medicina62061025

**Published:** 2026-05-25

**Authors:** Hwa-Yeon Lee, Yi-Heng Zhang, Ki-Choul Kim, Hyoung-Won Lim

**Affiliations:** 1 Department of Physical Therapy, Graduate School, Dankook University, Cheonan 31116, Republic of Korea; jul1201@naver.com; 2 Department of Orthopaedic Surgery, Dankook University Hospital, Cheonan 31116, Republic of Korea; puhoo73@hanmail.net; 3 Department of Physical Therapy, College of Health Sciences, Dankook University, Cheonan 31116, Republic of Korea

**Keywords:** modified clamshell exercise, gluteus medius, knee flexion angle, muscle activity

## Abstract

*Background and Objectives*: The gluteus medius plays a key role in hip stabilization and lower extremity alignment. However, the influence of knee flexion angle during the modified clamshell exercise on muscle activation patterns remains unclear. This study aimed to examine the effects of different knee flexion angles on gluteus medius activity and relative muscle activation patterns during the modified clamshell exercise. *Materials and Methods*: Thirty healthy women aged 20–30 years performed the modified clamshell exercise at three knee flexion angles (60°, 90°, and 110°). Muscle activity was recorded using surface electromyography and normalized to maximal voluntary isometric contraction (%MVIC). Relative muscle activation was assessed using muscle activation ratios (GMed/TFL, GMed/QL, GMed/Sar, and GMed/GMax). Differences among conditions were analyzed using repeated-measures ANOVA or the Friedman test, as appropriate. *Results*: Gluteus medius activation (*p* < 0.001, Kendall’s W = 0.57) and superior gluteus maximus activation (*p* < 0.001, ηp^2^ = 0.34) increased significantly with greater knee flexion angle, whereas tensor fasciae latae and quadratus lumborum activation decreased. The GMed/TFL (*p* < 0.001, ηp^2^ = 0.45) and GMed/QL (*p* < 0.001, Kendall’s W = 0.47) ratios also increased significantly, while the GMed/GMax ratio did not differ significantly. No meaningful differences were observed between 90° and 110°. *Conclusions*: Knee flexion angle influenced muscle activation patterns during the modified clamshell exercise. Compared with 60°, knee flexion angles of 90° and 110° were associated with greater gluteus medius activity and more favorable relative activation patterns; however, no additional benefit was observed beyond 90°. Given the low absolute activation levels, these findings reflect low-load motor control characteristics rather than strengthening effects. The results may be relevant for early-phase rehabilitation under controlled conditions, but their clinical applicability remains limited.

## 1. Introduction

Independent movement and the ability to perform activities of daily living are important components of health-related quality of life and functional well-being [1]. Efficient performance of these activities requires adequate lower extremity function, particularly optimal hip joint function [2]. When hip joint function is impaired, load distribution across joint surfaces may be altered, potentially leading to biomechanical abnormalities and clinical dysfunction [2,3]. Previous studies have suggested that such limitations are often associated with weakness of the hip abductor and external rotator muscles [3]. Therefore, identifying rehabilitation strategies that effectively enhance hip abductor muscle activity is essential for improving lower extremity function and clinical outcomes [4].

The primary muscles contributing to hip abduction include the gluteus medius (GMed), the superior portion of the gluteus maximus (GMax), and the tensor fasciae latae (TFL). These muscles differ in anatomical structure and fiber orientations but collectively contribute to lower extremity kinematics and pelvic stabilization [5,6]. Among them, the gluteus medius plays a central role in maintaining pelvic stability during weight-bearing activities and facilitating lower extremity movement [7]. Weakness of the gluteus medius has been associated with compensatory movement patterns, gait instability [8], and altered lower extremity alignment, including increased hip internal rotation and pelvic asymmetry [9]. These alterations may promote compensatory overactivation of the tensor fasciae latae, potentially leading to muscle imbalance and functional impairment [10,11], and have been linked to musculoskeletal disorders such as patellofemoral pain syndrome [12,13]. Accordingly, improving gluteus medius function and optimizing neuromuscular recruitment patterns during hip abduction are considered important objectives in rehabilitation [14,15].

Despite the importance of gluteus medius function, compensatory muscle activation frequently occurs during hip abductor exercises. In particular, excessive activation of the tensor fasciae latae and quadratus lumborum (QL) has been reported during attempts to increase gluteus medius activity [16]. When lumbopelvic stability is insufficient or the gluteus medius is weakened, other muscles may compensate, resulting in altered movement patterns [3]. Such compensatory activity may increase unnecessary lateral movement of the lumbar and pelvic regions, potentially elevating mechanical stress on the lumbar spine and contributing to low back pain [17]. Therefore, identifying exercise conditions that facilitate relatively gluteus medius activation while minimizing compensatory activity of surrounding muscles is clinically relevant. In addition, the gluteus medius and sartorius (Sar) have been suggested to contribute to pelvic stability, and coordination between these muscles may be associated with balance control during functional activities such as walking and running [18].

Various exercises have been proposed to improve gluteus medius function, including side-lying hip abduction, single-leg squats, and clamshell exercises [14,15]. Among these, side-lying hip abduction and clamshell-based exercises are open kinetic chain exercises commonly used in individuals who have difficulty performing weight-bearing activities [19]. The conventional clamshell exercise can be performed without specialised equipment; however, the modified clamshell exercise used in the present study required additional apparatus and examiner supervision to standardize movement execution and maintain lumbopelvic alignment. Localized exercises such as the modified clamshell exercise have been reported to promote relatively greater gluteus medius activation compared with multi-joint functional exercises and are therefore frequently used as foundational exercises in early rehabilitation [11,15]. In addition to muscle strengthening, these exercises may also facilitate neuromuscular re-education and coordination [20]. Surface electromyography (EMG) is widely used to quantify muscle activation patterns during therapeutic exercises and to evaluate relative muscle recruitment.

Previous studies have demonstrated that hip joint position can influence gluteus medius activation and modulate activation of the tensor fasciae latae during the modified clamshell exercise [11,15,21]. However, inconsistent findings have been reported regarding the optimal hip angle for maximizing gluteus medius activity, with some studies suggesting peak activation at 30°, while others report higher activation at 60° [22]. These discrepancies may reflect the complexity of the modified clamshell exercise, which involves combined hip abduction and external rotation and engages multiple muscles simultaneously [23,24]. Because the hip and knee joints function as part of an integrated kinetic chain, changes in knee joint position may influence hip muscle recruitment through biomechanical and neuromuscular interactions. Despite this relationship, the influence of knee flexion angle on muscle activation patterns and intermuscular coordination during the modified clamshell exercise has not been systematically investigated. Furthermore, the effect of knee flexion angle on compensatory activation of the tensor fasciae latae and quadratus lumborum remains unclear. Examining muscle activation ratios may provide additional insight into relative muscle recruitment patterns, although such measures should be interpreted with caution.

Therefore, the purpose of this study was to investigate the effects of knee flexion angle on muscle activation and relative activation patterns of key hip muscles during the modified clamshell exercise. Specifically, muscle activity of the gluteus medius, superior portion of the gluteus maximus, tensor fasciae latae, quadratus lumborum, and sartorius was compared across knee flexion angles of 60°, 90°, and 110°. We hypothesized that increasing knee flexion angle would be associated with increased activation of the GMed and superior Gmax, along with reduced activation of compensatory muscles such as the TFL and QL. We further hypothesized that greater knee flexion angles would be associated with changes in activation ratios, reflecting relative differences in muscle recruitment rather than definitive selective activation.

This study addresses a clinically relevant but under-investigated parameter—knee flexion angle—which is frequently modified in practice despite limited biomechanical evidence.

## 2. Materials and Methods

### 2.1. Participants

This study included 30 healthy adult women and employed a repeated-measures experimental design. Participants were recruited through voluntary participation. The inclusion criteria were as follows: no history of instability or musculoskeletal disorders in the lower extremities, including the hip and knee joints; no participation in resistance training or rehabilitation exercise programs for the lower extremities within the previous 3 months; no history of hip or knee surgery within the past 6 months; and no open wounds or inflammatory conditions at the electrode attachment sites. Participants who did not meet these criteria or who reported pain during the maximal voluntary isometric contraction (MVIC) measurements were excluded.

The sample size was determined using an a priori power analysis performed with G*Power software (version 3.1, Heinrich Heine University Düsseldorf, Düsseldorf, Germany). Based on a repeated-measures one-way ANOVA design with three levels, a significance level of α = 0.05, statistical power of 0.80, and an effect size of f = 0.25, the minimum required sample size was calculated to be 28. Considering potential data loss due to missing data or outliers, two additional participants were recruited, resulting in a total of 30 participants.

This study was conducted in accordance with ethical standards for human research. All participants were fully informed of the purpose, procedures, potential risks, and benefits of the study and provided written informed consent prior to participation. Participants were informed that they could withdraw from the study at any time without disadvantage, and all procedures complied with privacy protection regulations. The study was approved by the Institutional Review Board of Dankook University (IRB No. DKU 2024-12-015-003).

### 2.2. Measurement Tools and Methods

#### 2.2.1. Goniometer

Hip and knee joint angles were measured using a 360° stainless steel goniometer (Baseline^®^, Balance Body, Sialkot, Pakistan). For knee joint measurements the axis was aligned with the lateral tibiofemoral joint line, the stationary arm with the lateral thigh, and the moving arm with the longitudinal axis of the lower leg. For hip joint angle measurements, the axis was positioned over the greater trochanter, with the stationary arm aligned with the lateral trunk and the moving arm aligned with the thigh.

To standardize movement, each participant’s maximal hip abduction without visible pelvic compensation was first determined. A target bar was then fixed at this individualized position and maintained across all knee flexion conditions (60°, 90°, and 110°). To verify consistency, hip abduction angles were measured for each trial using a goniometer.

All measurements were performed by a single trained examiner using a standardized procedure to minimize variability. The examiner received prior training to ensure consistent identification of anatomical landmarks and angle measurement.

#### 2.2.2. Electromyography

A wireless surface electromyography system (Desk DTS EMG System, Noraxon Inc., Scottsdale, AZ, USA) was used to record muscle activity of the gluteus medius (GMed), superior gluteus maximus (GMax), tensor fasciae latae (TFL), quadratus lumborum (QL), and sartorius (Sar) during the modified clamshell exercise. Signals were sampled at 1024 Hz, band-pass filtered (20–450 Hz), and analyzed using MyoResearch software (version 1.8, Noraxon Inc., Scottsdale, AZ, USA).

Disposable Ag/AgCl electrodes (Blue Sensor, Medicotest, Denmark) were placed on the dominant limb. The dominant limb was selected to reduce inter-limb variability and improve measurement consistency in this repeated-measures design. Because all participants were healthy and asymptomatic, this approach was considered appropriate for experimental control.

**Figure 1:**
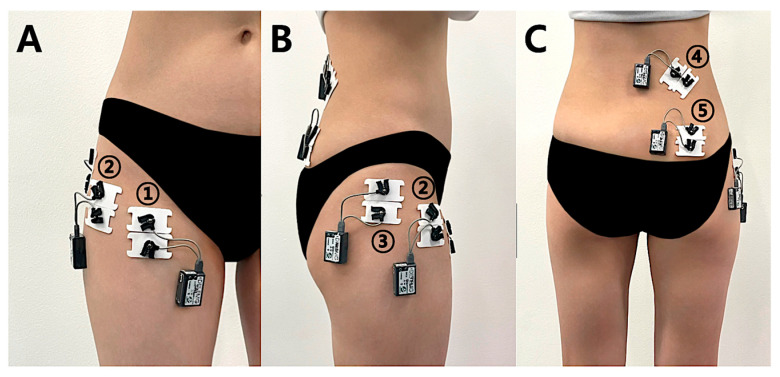
Surface EMG electrode placement. (**A**) Anterior view showing Sartorius (1) and Tensor fasciae latae (2); (**B**) Lateral view showing Tensor fasciae latae (2) and Gluteus medius (3); (**C**) Posterior view showing Quadratus lumborum (4) and superior portion of gluteus maximus (5).

Electrode placement followed established anatomical guidelines [25], with detailed locations shown in Figure 1. Active electrodes were placed with a 20 mm inter-electrode distance, and the ground electrode was positioned at a site that did not interfere with testing. EMG signals were transmitted, digitized, and stored on a laptop computer.

All measurements were performed by the same examiner. Pilot testing was conducted prior to data collection to improve procedural consistency.

#### 2.2.3. Maximal Voluntary Isometric Contraction (MVIC)

Maximal voluntary isometric contraction (MVIC) was measured for the GMed, GMax, TFL, QL, and Sar to normalize EMG signals recorded during the modified clamshell exercise. Muscle activity was expressed as a percentage of MVIC (%MVIC). The MVIC testing positions and procedures for each muscle were based on previous studies [11,19,25,26]. During testing, participants performed a 5 s maximal isometric contraction paced by a metronome set at 60 beats/min. Three trials were recorded for each muscle, with 10 s of rest between trials and 30 s of rest between measurements of different muscles. EMG signals were processed using the root mean square (RMS) method, and the mean RMS value obtained from the middle 3 s of each trial, excluding the first and last 1 s, was used as the reference value for 100% MVIC [26].

To assess intra-session reliability of EMG measurements, intraclass correlation coefficients (ICCs) were calculated for %MVIC values across the three repeated trials for each muscle under each knee flexion condition. ICC values were interpreted as follows: >0.90 excellent, 0.75–0.90 good, 0.50–0.75 moderate, and <0.50 poor reliability.

Muscle activation ratios were calculated to assess relative muscle activation patterns. Specifically, the ratios GMed/GMax, GMed/TFL, GMed/QL, and GMed/Sar were obtained by dividing the %MVIC of the gluteus medius by that of each corresponding muscle. These ratios were used to reflect relative differences in muscle recruitment rather than definitive selective activation.

### 2.3. Experimental Procedure

After completing the MVIC measurements, participants performed the modified clamshell exercise at three knee flexion angles (60°, 90°, and 110°). The order of the three knee flexion conditions was randomized using an online randomization generator (Random.org, https://www.random.org/). Each condition was repeated three times, with a 1 min rest period between trials to minimize muscle fatigue and a 10 min rest period between conditions [27].

In this study, the modified clamshell exercise was defined as a standardized variation of the traditional side-lying clamshell exercise. The protocol included a fixed hip flexion angle of 30°, controlled knee flexion angles (60°, 90°, and 110°), a target bar to standardize hip abduction range, and a pressure biofeedback unit to monitor lumbopelvic stability. These modifications were implemented to improve movement consistency and reduce compensatory trunk and pelvic motion.

Participants performed the exercise in a side-lying position with the dominant leg positioned at 30° of hip flexion and the ankle maintained in a neutral position [27,28]. The hip flexion angle was kept constant across all conditions, while only the knee flexion angle was varied to ensure consistent comparison and minimize compensatory motion [2,3].

The selected knee flexion angles were based on the functional range of motion commonly observed during daily activities (60–120°) [29]. Accordingly, three representative angles (60°, 90°, and 110°) were chosen to reflect shallow, typical, and deeper flexion positions, respectively [30].

Knee flexion angles were verified using a goniometer [31].

Hip abduction range was standardized using a target bar, which indicated the maximal abduction position achievable without loss of pelvic alignment [32]. To maintain lumbopelvic stability, a pressure biofeedback unit (Stabilizer Pressure Biofeedback, Chattanooga, DJO LLC., Lewisville, TX, USA) was placed under the lumbar region. Participants were instructed to maintain pressure between 35 and 45 mmHg throughout the exercise [33]. The examiner continuously monitored alignment and pressure and provided verbal feedback when necessary.

**Figure 2:**
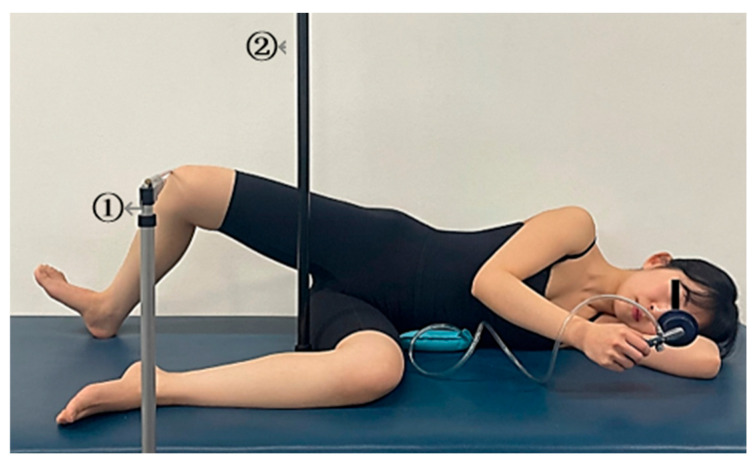
Target bar during Modified Clamshell Exercise. ① Knee target bar to provide visual feedback; ② Hip flexion target bar to maintain a constant 30° hip flexion.

Each repetition involved abducting the leg to the target position, holding the position for 5 s, and returning to the starting position [34]. Movements were paced using a metronome set at 60 beats/min (Figure 2).

### 2.4. Outcomes

The primary outcome variable of this study was gluteus medius activation expressed as %MVIC. This variable was selected to examine the effect of knee flexion angle on gluteus medius activity during the modified clamshell exercise.

Secondary outcomes included activation of the superior gluteus maximus, tensor fasciae latae, quadratus lumborum, and sartorius, as well as muscle activation ratios (GMed/GMax, GMed/TFL, GMed/QL, and GMed/Sar). These variables were analyzed to provide additional information on relative muscle activation patterns and potential compensatory muscle activity.

### 2.5. Statistical Analysis

Statistical analyses were performed using SPSS software (version 26.0; IBM Corp., Armonk, NY, USA). Participant characteristics were summarized as mean ± standard deviation.

Within-subject 95% confidence intervals were calculated using the Cousineau–Morey method for repeated-measures data.

The primary outcome was gluteus medius activation, and all other variables were treated as secondary outcomes.

Normality of muscle activity and activation ratio data was assessed using the Shapiro–Wilk test. Differences among the three knee flexion angles (60°, 90°, and 110°) were analyzed using one-way repeated-measures analysis of variance (ANOVA) for normally distributed variables and the Friedman test for non-normally distributed variables.

When significant main effects were identified, Bonferroni-adjusted post hoc tests or pairwise comparisons were performed as appropriate.

For muscle activity variables, repeated-measures ANOVA was applied to the superior gluteus maximus and quadratus lumborum, whereas the Friedman test was applied to the gluteus medius, tensor fasciae latae, and sartorius. For muscle activation ratio variables, repeated-measures ANOVA was applied to GMed/TFL, whereas the Friedman test was applied to GMed/GMax, GMed/QL, and GMed/Sar.

Effect sizes for repeated-measures ANOVA were expressed as partial eta squared (ηp^2^), with values of 0.01, 0.06, and 0.14 indicating small, medium, and large effects, respectively [35]. Kendall’s coefficient of concordance (W) was reported as the effect size for Friedman test results. Statistical significance was set at α = 0.05.

## 3. Results

### 3.1. Participant Characteristics

A total of 30 healthy adult women participated in this study. The mean age, height, and body weight were 26.3 ± 2.86 years, 161.4 ± 4.51 cm, and 53.7 ± 7.34 kg, respectively.

### 3.2. Muscle Activation by Knee Flexion Angle

The intraclass correlation coefficients (ICCs) for %MVIC values demonstrated good to excellent intra-session reliability across all measured muscles, ranging from 0.783 to 0.977. Detailed ICC values are presented in Table 1.

**Figure 3:**
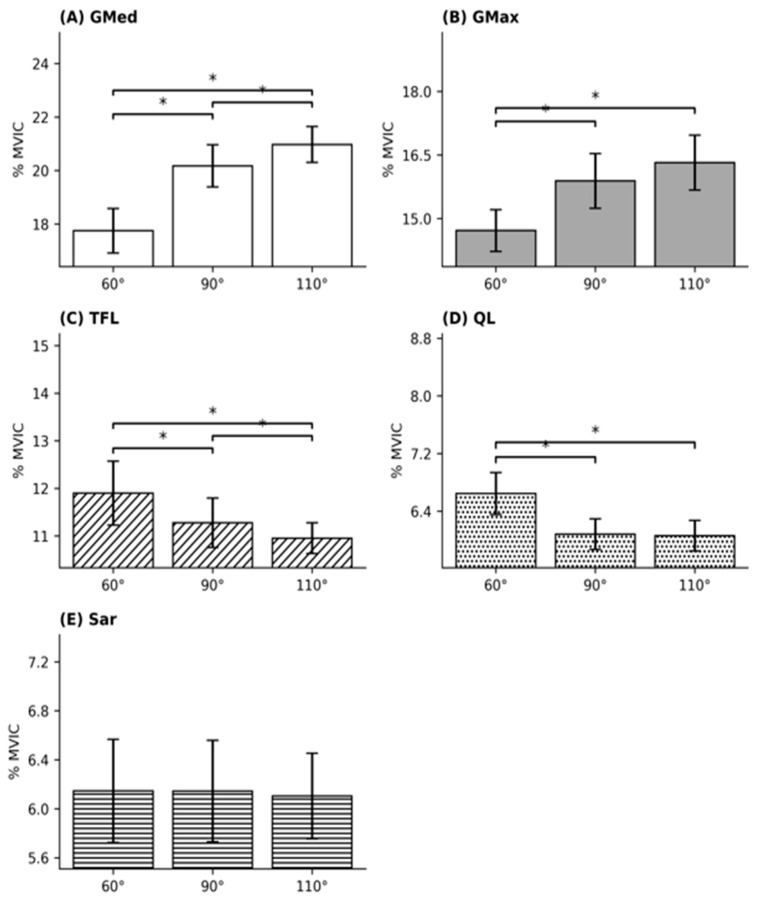
Muscle activation of the gluteus medius, superior gluteus maximus, tensor fasciae latae, quadratus lumborum, and sartorius during the modified clamshell exercise at knee flexion angles of 60°, 90°, and 110°. Error bars indicate standard deviation (SD). Significant differences between conditions are indicated by * *p* < 0.05; *Y*-axis scales differ across panels for clarity.

Muscle activation according to knee flexion angle is presented in Table 2 and Figure 3. The achieved hip abduction angles were 27.57 ± 1.74° at 60°, 29.21 ± 1.81° at 90°, and 30.14 ± 1.88° at 110°. No significant difference in achieved hip abduction angle was observed among the three knee flexion conditions (*p* = 0.105).

Gluteus medius activation differed significantly among the three conditions (χ^2^ = 34.20, *p* < 0.001, Kendall’s W = 0.57). Activation increased progressively with greater knee flexion angle, with significant differences among all three conditions (60° < 90° < 110°).

Superior gluteus maximus activation also differed significantly among conditions (F = 15.05, *p* < 0.001, ηp^2^ = 0.34). Post hoc analysis showed that activation at 60° was significantly lower than at 90° and 110°, whereas no significant difference was observed between 90° and 110°.

Tensor fasciae latae activation differed significantly among conditions (χ^2^ = 32.47, *p* < 0.001, Kendall’s W = 0.54). Activation decreased progressively as knee flexion angle in-creased, with significant differences among all three conditions (60° > 90° > 110°).

Quadratus lumborum activation also differed significantly among conditions (F = 8.82, *p* < 0.001, ηp^2^ = 0.23). Post hoc analysis showed that activation at 60° was significantly higher than at 90° and 110°, whereas no significant difference was observed between 90° and 110°.

Sartorius activation did not differ significantly among the three conditions (χ^2^ = 2.40, *p* = 0.301, Kendall’s W = 0.04).

### 3.3. Muscle Activation Ratios by Knee Flexion Angle

**Figure 4:**
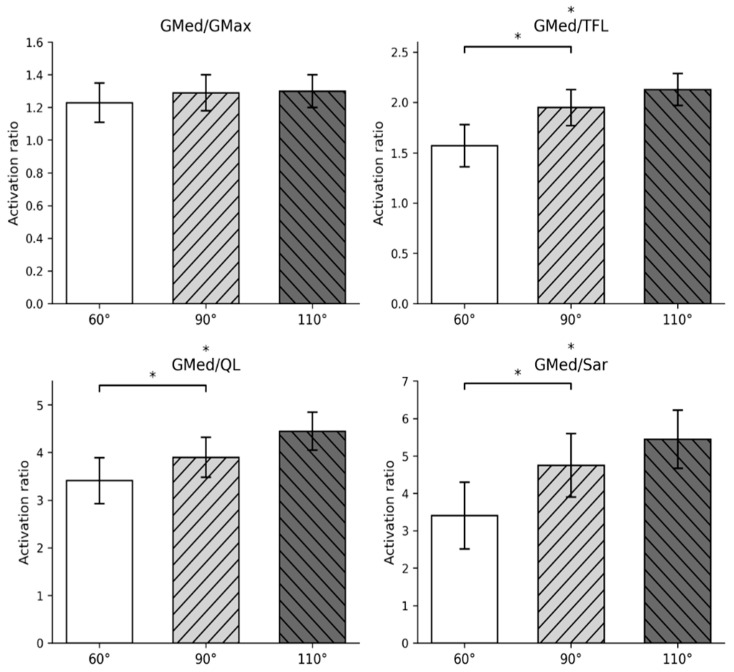
Muscle activation ratios during the modified clamshell exercise at knee flexion angles of 60°, 90°, and 110°. Values are presented as mean ± within-subject 95% confidence intervals, adjusted for repeated measures by removing between-subject variability. Significant differences between conditions are indicated by * *p* < 0.05. *Y*-axis scales differ across panels for clarity.

Muscle activation ratios according to knee flexion angle are presented in Table 3 and Figure 4.

The GMed/TFL activation ratio was 1.57 ± 0.54 at 60°, 1.95 ± 0.77 at 90°, and 2.13 ± 0.72 at 110°, and differed significantly among the three conditions (F = 23.34, *p* < 0.001, η^2^ = 0.45). Bonferroni-adjusted post hoc analysis indicated that the ratios at 90° and 110° were significantly higher than the ratio at 60°, whereas no significant difference was observed between 90° and 110°.

Similarly, the GMed/QL activation ratio differed significantly among the three conditions (χ^2^ = 28.47, *p* < 0.001, W = 0.47). Bonferroni-adjusted pairwise comparisons showed that the ratios at 90° and 110° were significantly higher than the ratio at 60°, whereas no significant difference was observed between 90° and 110°.

In contrast, the GMed/GMax activation ratio did not differ significantly among the three conditions (χ^2^ = 1.26, *p* = 0.290, W = 0.04).

The GMed/Sar activation ratio was 3.41 ± 1.80 at 60°, 4.74 ± 3.54 at 90°, and 5.45 ± 5.40 at 110°, and differed significantly among the three conditions (χ^2^ = 12.60, *p* = 0.001, W = 0.21). Bonferroni-adjusted pairwise comparisons indicated that the ratios at 90° and 110° were significantly higher than the ratio at 60°, whereas no significant difference was observed between 90° and 110°.

## 4. Discussion

This study examined the effect of knee flexion angle on gluteus medius activation and relative muscle activation patterns during the modified clamshell exercise. The main finding was that increasing knee flexion angle was associated with greater gluteus medius activation and reduced activation of the tensor fasciae latae and quadratus lumborum, resulting in more favorable relative activation patterns. However, no meaningful differences were observed between 90° and 110°, suggesting that angles of 90° or greater may be sufficient under controlled conditions.

These findings should be interpreted in light of the study population. Only healthy young women were included to reduce variability related to sex-specific morphology and biomechanical characteristics, which may influence muscle activation patterns. Women generally exhibit greater pelvic width, femoral shaft angle, femoral anteversion, and Q-angle than men [36,37]. Therefore, extrapolation to other populations, particularly individuals with musculoskeletal disorders, should be made with caution.

A possible explanation for the observed increase in gluteus medius activation with greater knee flexion angle is the alteration of external mechanical demand due to changes in distal limb configuration. Because hip abduction range was standardized across conditions, differences in joint excursion are unlikely to explain the results. Instead, increased knee flexion may have modified the gravitational moment relative to the hip joint, requiring greater internal hip abductor torque [2]. However, as joint moments and segmental mechanics were not directly measured, this interpretation should be considered a biomechanical hypothesis rather than a confirmed mechanism.

Similarly, the reduction in tensor fasciae latae activation at greater knee flexion angles may be explained by changes in the length–tension relationship. The tensor fasciae latae is influenced by both hip and knee position through its connection to the iliotibial tract [5,6]. Increased knee flexion may place the muscle in a relatively shortened position, potentially reducing its force-generating capacity due to suboptimal length–tension conditions [38]. This may shift the relative contribution toward the gluteus medius during hip abduction. However, this explanation remains speculative because muscle length and force production were not directly measured.

Although statistically significant differences were observed, the absolute magnitude of change in gluteus medius activation was small, and overall activation levels remained low. These values fall below commonly cited thresholds for strength adaptation [39]. Therefore, the modified clamshell exercise, as performed in this study, should be interpreted as a low-load motor control task rather than a strengthening intervention [11,15,19].

Within this context, increasing knee flexion angle may be associated with modest improvements in relative muscle activation patterns, as reflected by increases in the GMed/TFL and GMed/QL ratios. However, these ratios should be interpreted as indicators of relative muscle activity rather than definitive evidence of selective activation. In contrast, the lack of change in the GMed/GMax ratio may reflect the cooperative role of these muscles during hip abduction and external rotation [15,24]. The increase in the GMed/Sar ratio should be interpreted cautiously, as the sartorius is not a primary compensatory muscle in this movement [40].

An important consideration is that the exercise protocol was performed under highly standardized and apparatus-dependent conditions, including the use of a target bar, pressure biofeedback, and continuous examiner supervision. These conditions differ from typical clinical or home-based exercise environments, where such controls are not available. Therefore, the present findings may not directly translate to less controlled settings, and their external validity remains limited.

Taken together, these results provide confirmatory biomechanical evidence supporting the commonly used 90° knee flexion position during the modified clamshell exercise [11,15]. The findings suggest that increasing knee flexion beyond 90° does not confer additional benefit under controlled conditions. Importantly, the results should be interpreted as reflecting relative changes in muscle recruitment during low-load exercise, and their clinical impact remains uncertain. Whether these differences translate into meaningful functional or clinical outcomes requires further investigation in symptomatic populations.

Several limitations should be considered. First, this study included a relatively small and homogeneous sample of healthy young adult women, and all measurements were performed on the dominant limb. Therefore, the findings may not be generalizable to males, other age groups, individuals with different physical activity levels, or patients with musculoskeletal disorders, particularly those with side-specific dysfunction.

Second, only immediate muscle activation responses were assessed, and long-term neuromuscular adaptations were not evaluated.

Third, surface electromyography has inherent limitations, including the inability to assess deep muscles and the potential for cross-talk from adjacent muscles.

Fourth, although lumbopelvic stability was controlled using a pressure biofeedback unit, pressure values and compliance were not quantitatively recorded. In addition, intra-rater reliability of goniometric measurements was not formally assessed. Therefore, measurement-related variability cannot be completely excluded.

Finally, participants’ physical activity levels were not directly assessed, and multiple secondary outcomes were analyzed. These factors may have influenced muscle activation patterns and increase the possibility of type I error. Therefore, findings related to secondary variables should be interpreted with caution.

In addition, although hip abduction range of motion did not differ significantly between conditions, a small directional increase was observed with greater knee flexion angles. Therefore, a potential contribution of this trend to the observed differences in muscle activation cannot be entirely ruled out.

Future studies should include more diverse populations, assess long-term training effects, and incorporate complementary methods such as kinematic or kinetic analyses.

## 5. Conclusions

Under controlled conditions in healthy young women, knee flexion angles of 90° and 110° were associated with greater gluteus medius activation than 60°, with no meaningful difference between 90° and 110°. These findings provide confirmatory biomechanical support for the commonly used 90° knee flexion position and suggest that greater knee flexion does not confer additional benefit.

However, given the small magnitude of differences and the low overall activation levels, the modified clamshell exercise should be interpreted as a low-load motor control task rather than a strengthening intervention. Accordingly, these findings are most relevant to early-phase rehabilitation and neuromuscular re-education under supervised conditions.

Because the exercise protocol was performed under standardized and apparatus-dependent conditions, caution is required when applying these findings to typical clinical or home-based settings. Therefore, the present results should be considered preliminary biomechanical evidence, and further studies are needed to determine their applicability in individuals with musculoskeletal dysfunction.

## Figures and Tables

**Table 1:** Intra-session reliability (ICC) of %MVIC values across knee flexion conditions.

Muscle	60°	90°	110°
Gluteus medius	0.797	0.835	0.789
Superior gluteus maximus	0.881	0.896	0.783
TFL	0.798	0.893	0.920
Quadratus lumborum	0.932	0.896	0.920
Sartorius	0.947	0.966	0.977

**Table 2:** Comparisons of muscle activation during the modified clamshell exercise according to knee flexion angle (%MVIC).

Muscle	Angle 60°	Angle 90°	Angle 110°	Test Statistic	*p*	Effect Size (ηp^2^ or W)	Post Hoc
GMed	17.67 ± 5.61	20.08 ± 5.75	21.17 ± 5.69	χ^2^ = 34.20	<0.001 *	W = 0.57	a < b < c
GMax	14.66 ± 3.90	15.95 ± 4.90	16.61 ± 3.83	F = 15.05	<0.001 *	ηp^2^ = 0.34	a < b, c
TFL	11.90 ± 3.53	11.26 ± 4.05	10.73 ± 3.69	χ^2^ = 32.47	<0.001 *	W = 0.54	a > b > c
QL	6.56 ± 3.03	6.07 ± 2.87	5.95 ± 2.83	F = 8.82	<0.001 *	ηp^2^ = 0.23	a > b, c
Sar	6.15 ± 3.08	6.13 ± 4.27	6.10 ± 3.97	χ^2^ = 2.40	0.301	W = 0.04	-

GMed, gluteus medius; GMax, superior gluteus maximus; TFL, tensor fasciae latae; QL, quadratus lumborum; Sar, sartorius; %MVIC, percent maximal voluntary isometric contraction; ηp^2^, partial eta squared; W, Kendall’s coefficient of concordance. Repeated-measures ANOVA was used for the normally distributed variables, whereas the Friedman test was used for variables that violated the normality assumption. Bonferroni-adjusted paired *t*-tests or Wilcoxon signed-rank tests were used for post hoc comparisons, as appropriate. Statistical significance was set at *p* < 0.05, and *p* < 0.017 was considered significant for post hoc comparisons following the Friedman test. * *p* < 0.05. a = angle 60°, b = angle 90°, c = angle 110°.

**Table 3:** Comparison of muscle activation ratios during the modified clamshell exercise according to knee flexion angle.

Ratio	Angle 60°	Angle 90°	Angle 110°	Test Statistic	*p*	Effect Size (ηp^2^ or W)	Post Hoc
GMed/GMax	1.24 ± 0.35	1.30 ± 0.32	1.29 ± 0.26	χ^2^ = 1.26	0.290	W = 0.04	-
GMed/TFL	1.57 ± 0.54	1.95 ± 0.77	2.13 ± 0.72	F = 23.34	<0.001 *	ηp^2^ = 0.45	a < b, c
GMed/QL	3.21 ± 1.49	3.92 ± 1.49	4.37 ± 2.62	χ^2^ = 28.47	<0.001 *	W = 0.47	a < b, c
GMed/Sar	3.41 ± 1.80	4.74 ± 3.54	5.45 ± 5.40	χ^2^ = 12.60	<0.001 *	W = 0.21	a < b, c

ηp^2^, partial eta squared; W, Kendall’s coefficient of concordance. Repeated-measures ANOVA was used for the normally distributed variable (GMed/TFL), whereas the Friedman test was used for variables that violated the normality assumption (GMed/GMax, GMed/QL, and GMed/Sar). Post hoc analyses were performed using Bonferroni-adjusted paired *t*-tests or Wilcoxon signed-rank tests with Bonferroni correction, as appropriate. Statistical significance was set at *p* < 0.05, and *p* < 0.017 was considered significant for post hoc comparisons following the Friedman test. * *p* < 0.05. a = angle 60°, b = angle 90°, c = angle 110°.

## Data Availability

Original data are available from the corresponding author upon reasonable request.
